# The Influence of Type of Maternity Care Provider on Early Breastfeeding Rates. A Retrospective Cohort Study of All Women Who Gave Birth in Victoria, Australia 2010 to 2020

**DOI:** 10.1111/ajo.70191

**Published:** 2026-10-28

**Authors:** Maja Johnsson, Sophia Holmlund, Isabelle Augustsson, Amanda Salander, Christine E. East, Lisa H. Amir, Kristina Edvardsson

**Affiliations:** ^1^ Department of Clinical Sciences, Division of Obstetrics and Gynecology Umea University Umea Sweden; ^2^ Department of Nursing Umea University Umea Sweden; ^3^ Judith Lumley Centre, School of Nursing and Midwifery La Trobe University Melbourne Australia; ^4^ Mercy Health Melbourne Victoria Australia; ^5^ Breastfeeding Service Royal Women's Hospital Parkville Victoria Australia

## Abstract

**Background:**

Breastfeeding plays a vital role in infant health and development. Maternity care providers are well‐positioned to support breastfeeding during the postnatal period. In Australia, there are multiple options for public and private maternity care. To date, there is limited information on how caregiver and hospital type influence early breastfeeding.

**Aim:**

To examine associations between type of care provider during antenatal and intrapartum care, hospital type and early breastfeeding rates in Victoria, Australia.

**Methods:**

Retrospective cohort study of all live, singleton, term births in Victoria, Australia 2010–2020 (*N* = 638,809). We investigated associations between variables using bivariate and multivariable logistic regression models.

**Results:**

Most women (95.7%) initiated breastfeeding, and 77.1% provided the last feed before hospital discharge exclusively from the breast. About a quarter of infants (27.0%) received commercial milk formula while in hospital. Women who received antenatal or intrapartum care from a midwife or general practitioner were more likely to initiate breastfeeding, and their infants to have their last feed before discharge from the breast, and less likely to receive formula in hospital compared to those cared for by an obstetrician. Dyads who received care in public hospitals had better breastfeeding outcomes than those in private hospitals. The small proportion (0.2%) who received private midwifery care showed the most favourable breastfeeding outcomes.

**Conclusions:**

Early breastfeeding outcomes in Victoria differ in relation to antenatal and intrapartum care provider and hospital type, and indicate disparities in supportive breastfeeding practices. Implementation of Baby Friendly Health procedures may help standardise care across these settings.

## 1 Introduction

Breastfeeding plays an important role in promoting the health of mothers and infants [1]. The World Health Organisation (WHO) recommends that breastfeeding is initiated within the first hour of birth and that infants are exclusively breastfed for the first 6 months of life [2]. Following that, continuous breastfeeding, in conjunction with the introduction of complementary foods, is recommended up to 2 years of age or beyond [2].

Materntity care providers have an important role in promoting maternal and infant health and healthy postnatal practices, including providing breastfeeding support.

To improve healthcare for infants, their mothers and families, the WHO and the United Nations Children's Fund (UNICEF) introduced the Baby Friendly Hospital Initiative (BFHI) in 1991 [3]. Adherence to the *Ten Steps to Successful Breastfeeding* has been shown to improve breastfeeding outcomes worldwide [4]. In Australia, maternity services may apply for BFHI accreditation, and if they meet the criteria, the accreditation is valid for a three‐year period, after which renewal is required [5]. In 2023, 21.4% of Australian maternity facilities were accredited under the Baby Friendly Health Initiative [3].

Early supplementation with commercial milk formula has been linked to shorter duration of breastfeeding, which in turn is linked to suboptimal health outcomes for mother and child [6, 7]. It is therefore important to reduce unnecessary use of formula in hospitals, and the BFHI guidelines state that formula should only be introduced when medically indicated or if it is the parents' informed choice [5]. The medical indications for supplementation of healthy, term infants are summarised in Box 1.

BOX 1 **Possible medical indications for supplementation in healthy term infants (37–42 weeks) according to BFHI Australia [5].**

*Infant indications*

- Hypoglycaemia.
- Clinical or laboratory evidence of significant dehydration.
- Significant weight loss may be an indication of inadequate milk transfer or low milk production, but a thorough evaluation of infant feeding is required before automatically ordering supplementation.
- Delayed or inadequate bowel movements or continued meconium stools on day 5 may be an indication of inadequacy of breastfeeding.
- Hyperbilirubinemia associated with poor breast milk intake despite appropriate intervention and marked by ongoing weight loss and limited stooling.
- Macronutrient supplementation is indicated, such as for the rare infant with inborn errors of metabolism.

*Maternal indications*

- Delayed secretory activation with signs of inadequate intake by the infant.
- Primary glandular insufficiency.
- Breast pathology or prior breast surgery resulting in poor milk production.
- Certain maternal medications (e.g., chemotherapy, psychotherapeutic drugs, anti‐epileptic drugs, long‐lasting radioactive compounds).
- Intolerable pain during feeding unrelieved by interventions.
- Severe illness that prevents a mother caring for her infant, for example, sepsis.
- Uncommon maternal conditions (e.g., HSV lesions on the breast, HCV positive and bleeding nipples, HIV positive using guidelines based on CD4 count and ART prophylaxis).

In Australia, maternity services can be accessed through different public and private ‘models of care’, differing in type of primary care provider, level of continuity, and the setting in which care is provided [8]. All citizens are entitled to the universal healthcare system, Medicare, and the public hospital system [9]. Some choose additional private health insurance, providing more options for healthcare, with out‐of‐pocket payments [10, 11].

Public hospital maternity care is the most common model of antenatal care (ANC), with 57% of maternity services providing at least one such model of care [8]. Common features of this model include that care is provided by midwives in collaboration with doctors, ANC is provided in onsite or outreach clinics, and intrapartum and postnatal care is provided in public hospitals [8]. Women attending a public hospital for overall maternity care may also access ANC in a Midwifery Group Practice or shared care between the hospital and the General Practitioner (GP), or a privately practising midwife [8].

In nearly half (49%) of all models, a public midwife is the lead maternity carer, coordinating care throughout pregnancy, birth, and the postnatal period. The second most common is to have a public specialist obstetrician (15%), followed by a shared arrangement (14%) [8]. The extent of continuity of carer varies across the different models. Full continuity is most common in models of care classified as *midwifery group practice caseload care, private midwifery care*, and *private obstetrician specialist care* [8].

To date, there is limited understanding of how early breastfeeding initiation and exclusivity are influenced by type of care provider during antenatal and intrapartum phases and hospital type. We therefore sought to investigate these associations.

## 2 Material and Methods

### 2.1 Study Design

This study involved a register‐based retrospective cohort study of 638,809 live, singleton, term births, using perinatal data obtained from the Victoria Perinatal Data Collection (VPDC).

### 2.2 Setting and Participants

The VPDC comprises information on all births (live or stillbirth) of at least 20 weeks' gestation (or 400 g birth weight in the absence of known gestational age) in Victoria, Australia. Birth data are reported to the VPDC by health services within 30 days following hospital discharge, or by the midwives/attending healthcare provider when a birth occurs outside of a health service [12]. Two validation studies of VPDC data have demonstrated high levels of accuracy between the data collection items and medical records [13, 14]. However, this does not account for potential discrepancies between actual infant feeding practices and their documentation on the medical records. The VPDC includes a wide range of data related to obstetric conditions, procedures, and outcomes. We used data on births for the period January 2010 to December 2020. We excluded all stillbirths, twins or multiple births, births < 37 weeks gestation, cases where the mother or the infant was admitted to a High Dependency Unit/Intensive Care Unit or Special Care Nursery/Neonatal Intensive Care Unit, and infants with congenital anomalies, since these factors are known to influence early breastfeeding. This gave us an analytical sample of 638,809 births.

### 2.3 Outcomes

Three outcome variables were examined: if breastfeeding was initiated (attempted to breastfeed the baby or express breast milk at least once); if infant formula was given in hospital (any infant formula given in hospital whether by bottle, cup, gavage, or other means); if the last feed before discharge was taken entirely from the breast (with no complementary feeding of any kind) [15].

### 2.4 Exposures

The independent variables included the discipline of ANC provider (obstetrician, midwife, GP, or other/no ANC provider); the discipline of lead intrapartum carer (obstetrician, midwife, GP, or other/no intrapartum care provider); and hospital type (referring to hospital type at birth: private, public or assisted by a private midwife).

### 2.5 Covariates/Confounders

Sociodemographic variables available in the VPDC dataset that are known to influence a range of health outcomes were included. The analyses were adjusted with the following covariates: age, marital status, world region of birth of mother, Indigenous status, parity, rurality, Index of Relative Socio‐Economic Disadvantage [IRSD] [16], smoking, method of birth, length of hospital stay, and Body Mass Index (BMI). A detailed description of the covariates can be found in Appendix 1.

### 2.6 Missing Values

Several variables had missing data. Ranging from less than 0.1% (maternal age) to 1.4% (relationship status). The outlier was BMI, with 5.9% missing.

### 2.7 Statistical Analyses

In addition to descriptive statistics, we undertook bivariate and multivariable logistic regression analyses to explore relationships between variables, and presented these as crude Odds Ratios (OR) and adjusted Odds Ratios (aOR) with their 95% Confidence Intervals (CI). Missing data were handled using complete case analysis. Of the 638,809 births in the analytical sample, 89.3%–90.5% had complete data for all covariates and were included in the adjusted regression analyses. All analyses were undertaken in IBM SPSS version 29 [17].

### 2.8 Ethical Considerations

Ethical approval was given by the La Trobe University Ethics committee (reference HEC22090). The Consultative Council on Obstetric and Paediatric Mortality and Morbidity (CCOPMM) in Victoria, Australia, approved use of VPDC data.

## 3 Results

### 3.1 Characteristics of the Population

There were 856,013 registered births in Victoria during the period January 2010 to December 2020. After applying our exclusion criteria, 638,809 births remained in our analytical sample. Table 1 presents sociodemographic, breastfeeding, and maternity care characteristics of the population.

**TABLE 1:** Socio‐demographic and pregnancy history characteristics of the women who gave birth in Victoria, 2010–2020 (*n*[%]).

Characteristics		*n*	%
Maternal age, years, *n =* 638 571	< 20	10 554	1.7
	20–24	62 826	9.8
	25–29	166 361	26.1
	30–34	236 030	37.0
	35–39	133 398	20.9
	40+	29 402	4.6
Marital status, *n* = 629 715	In a couple relationship	557 862	88.6
	Not in a couple relationship	71 853	11.4
Maternal world region of birth, *n* = 635 144	Australia	400 041	63.0
	Southern & Central Asia	65 530	10.3
	South‐East Asia	42 202	6.6
	North‐East Asia	31 802	5.0
	North Africa & the Middle East	22 647	3.6
	North‐West Europe	19 157	3.0
	Oceania & Antarctica	18 359	2.9
	Sub‐Saharan Africa	13 881	2.2
	Southern & Eastern Europe	12 413	2.0
	Americas	9112	1.4
Indigenous status, *n* = 635 659	Non‐indigenous	627 755	98.8
	Aboriginal or Torres Strait Islander origin	7904	1.2
Parity, *n = 638 756*	0	273 424	42.8
	1	234 526	36.7
	2	87 136	13.6
	3+	43 670	6.8
Area of living, *n* = 638 435	Major Cities of Australia	437 663	68.6
	Inner Regional Areas	158 637	24.8
	Outer Regional + Remote + Very Remote	42 135	6.6
Index of Relative Socio‐Economic Disadvantage (IRDS), *n* = 637 689	1 (most disadvantage)	90 663	14.2
	2	103 801	16.3
	3	137 236	21.5
	4	148 330	23.3
	5 (least disadvantage)	157 659	24.7
Smoking before gestational age 20 weeks, *n* = 631 037	No smoking at all before 20 weeks of pregnancy	574 662	91.1
	Quit smoking during pregnancy (before 20 weeks)	12 495	2.0
	Continued smoking before 20 weeks of pregnancy	43 880	7.0
BMI^a^, *n* = 600 921	< 18.5 Underweight	17 750	3.0
	18.5–24.9 Normal weight	307 420	51.2
	25.0–29.9 Pre‐obesity	160 876	26.8
	30.0–34.9 Obesity Class I	70 321	11.7
	35.0–39.9 Obesity Class II	28 325	4.7
	≥ 40 Obesity Class III	16 229	2.7
Method of birth, *n* = 638 774	Unassisted vaginal birth	344 339	53.9
	Vacuum extraction	48 969	7.7
	Forceps	46 794	7.3
	Planned caesarean—no labour	105 807	16.6
	Planned caesarean—labour	4480	0.7
	Unplanned caesarean—no labour	17 681	2.8
	Unplanned caesarean—labour	70 704	11.1
Length of hospital stay^b^, *n* = 637 144	Mean value: 2.66 days		
	< 1 day	24 135	3.8
	1 day	99 650	15.6
	2 days	198 608	31.2
	3 days	135 492	21.3
	4–6 days	176 501	27.7
	7+ days	2785	0.4
Breastfeeding characteristics			
Breastfeeding initiated^c^, *n* = 638 075	Yes	610 448	95.7
	No	27 627	4.3
Formula given in hospital^d^, *n* = 630 819	No	460 758	73.0
	Yes	170 061	27.0
Last feed before discharge^e^, *n* = 634 618	Taken entirely from breast	489 098	77.1
	Not taken entirely from breast	145 520	22.9
Maternity care characteristics			
Discipline of antenatal care provider^f^, *n* = 636 620	Obstetrician	323 649	50.8
	Midwife	220 416	34.6
	General practitioner	90 347	14.2
	No antenatal care provider or other care provider	2208	0.3
Discipline of lead intrapartum care provider^g^, *n* = 638 718	Obstetrician	393 434	61.6
	Midwife	204 687	32.0
	General practitioner	37 559	5.9
	No intrapartum care provider or other care provider	3038	0.5
Hospital type, *n* = 636 568	Public	490 694	77.1
	Private	144 380	22.7
	Private Midwife	1494	0.2

^a^ WHO classification.

^b^ Length of hospital stay—mother (postnatal).

^c^ Coded as Yes (attempted to breastfeed or express breastmilk) or No (did not attempt to breastfeed or express breastmilk).

^d^ Coded as Formula given (either by cup, bottle, gavage, or other means) in the hospital or Formula not given in the hospital.

^e^ Coded as Taken entirely from the breast (includes when the baby's entire last feed before discharge was taken directly from the breast) or Not taken entirely from the breast (includes when the baby's last feed before discharge involved any expressed breast milk or formula, administered via cup, spoon, gavage or other methods).

^f^ The discipline of the clinician who provided most occasions of antenatal care (VPDC manual).

^g^ The discipline of the clinician who, at the time of admission for the birth, is expected to be primarily responsible for making decisions regarding intrapartum care (VPDC manual).

#### 3.1.1 Sociodemographic Characteristics

The most common age group was 30–34 years (37.0%), the majority were in a couple relationship (88.6%) and born in Australia (63.0%). Among those born outside of Australia, the most common region was Southern and Central Asia (10.3%). A small minority identified as Indigenous (1.2%). The largest group of women had their first birth (42.8%). Two thirds of women lived in major cities of Australia (68.6%), and almost one quarter of the women belonged to the least disadvantaged group (IRSD Group 5) (24.7%). The majority did not smoke prior to 20 weeks of pregnancy (91.1%) and about half of women had normal BMI (51.2%).

#### 3.1.2 Breastfeeding Characteristics

The vast majority of women initiated breastfeeding (95.7%). Just over a quarter of the infants received formula in hospital (27.0%), and most women gave the last feed before discharge from the hospital entirely from the breast (77.1%).

#### 3.1.3 Maternity Care Characteristics

A slight majority of women received care from obstetricians during both the antenatal and intrapartum phases (50.8% and 61.6% respectively). The second most common lead care provider was a midwife for both phases (34.6% and 32.0% respectively). Just over two thirds of women (77.1%) received care in a public hospital, and nearly one quarter (22.7%) in a private hospital. Only a small proportion received care through a private midwife (0.2%). Over half (53.9%) of women had an unassisted vaginal birth, with a mean length of hospital stay of 2.6 days.

### 3.2 Association Between Caregiver and Early Breastfeeding Rates

#### 3.2.1 Association Between Discipline of Antenatal Care Provider and Early Breastfeeding

The findings summarised in Table 2 indicate that women who had a midwife (aOR 1.26, 95% CI 1.22–1.30) or a GP (aOR 1.25, 95% CI 1.21–1.31) as ANC provider were more likely to initiate breastfeeding compared to women who had an obstetrician as ANC provider. Women who had a midwife (aOR 0.78, 95% CI 0.77–0.79) or GP (aOR 0.74, 95% CI 0.72–0.76) as ANC provider had lower adjusted odds of the infant receiving formula in hospital, and higher adjusted odds of giving the last feed before discharge entirely from the breast (midwife: aOR 1.23, 95% CI 1.21–1.25; GP: aOR 1.39, 95% CI 1.36–1.42).

**TABLE 2:** Associations between care provider and early breastfeeding rates.

Care provider	Breastfeeding initiated^d^, Yes (%)/No (%)	Early breastfeeding rates					
		Crude OR			Adjusted OR^a^		
		Breastfeeding initiated^d^					
		cOR	CI 95%	*p*	aOR	CI 95%	*p*
**Antenatal care provider** ^b^							
Obstetrician	308 486 (95.5%)/14 648 (4.5%)	Ref.			Ref.		
Midwife	211 800 (96.2%)/8 460 (3.8%)	1.19	1.16–1.22	**< 0.001**	1.26	1.22–1.30	**< 0.001**
General Practitioner (GP)	86 168 (95.4%)/4 123 (4.6%)	0.99	0.96–1.03	0.67	1.26	1.21–1.31	**< 0.001**
No/other intrapartum care provider	1948 (88.4%)/256 (11.6%)	0.36	0.32–0.41	**< 0.001**	0.78	0.65–0.93	0.007
**Lead intrapartum care provider**							
Obstetrician	376 193 (95.8%)/16 645 (4.2%)	Ref.			Ref.		
Midwife	195 804 (95.7%)/8 768 (4.3%)	0.99	0.96–1.02	0.374	1.15	1.12–1.19	**< 0.001**
General Practitioner (GP)	35 486 (94.5%)/2 055 (5.5%)	0.76	0.73–0.80	**< 0.001**	1.28	1.21–1.35	**< 0.001**
No/other intrapartum care provider	2 881 (94.9%)/155 (5.1%)	0.82	0.70–0.97	0.018	1.30	1.09–1.56	0.**004**
**Hospital type**							
Public	467 219 (95.3%)/23 089 (4.7%)	Ref.			Ref.		
Private	139 551 (96.9%)/4 489 (3.1%)	1.54	1.49–1.59	**< 0.001**	0.65	0.62–0.68	**< 0.001**
Private midwife	1476 (99.2%)/12 (0.8%)	6.08	3.44–10.73	**< 0.001**	5.75	2.98–11.10	**< 0.001**

	Formula given in hospital,^e^ Yes (%)/No (%)	Formula given in hospital^e^					
		cOR	CI 95%	*p*	aOR	CI 95%	*p*
**Antenatal care provider** ^b^							
Obstetrician	103 239 (32.3%)/216 174 (67.7%)	Ref.			Ref.		
Midwife	46 998 (21.5%)/171 241 (78.5%)	0.58	0.57–0.58	**< 0.001**	0.78	0.77–0.79	**< 0.001**
General Practitioner (GP)	18 564 (20.9%)/70 279 (79.1%)	0.55	0.54–0.56	**< 0.001**	0.74	0.72–0.76	**< 0.001**
No/other intrapartum care provider	669 (30.7%)/1511 (69.3%)	0.93	0.85–1.02	0.104	0.91	0.82–1.03	0.126
**Lead intrapartum care provider**							
Obstetrician	120 782 (31.1%)/267 729 (68.9%)	Ref.			Ref.		
Midwife	41 280 (20.4%)/161 246 (79.6%)	0.57	0.56–0.58	**< 0.001**	0.86	0.84–0.87	**< 0.001**
General Practitioner (GP)	7470 (20.4%)/29 226 (79.6%)	0.57	0.55–0.58	**< 0.001**	0.70	0.68–0.72	**< 0.001**
No/other intrapartum care provider	505 (16.8%)/2497 (83.2%)	0.45	0.41–0.49	**< 0.001**	0.71	0.64–0.79	**< 0.001**
**Hospital type**							
Public	118 338 (24.4%)/366 097 (75.6%)	Ref.			Ref.		
Private	51 219 (35.9%)/91 491 (64.1%)	1.73	1.71–1.75	**< 0.001**	1.34	1.32–1.37	**< 0.001**
Private midwife	8 (0.6%)/1446 (99.4%)	0.02	0.01–0.03	**< 0.001**	0.02	0.01–0.07	**< 0.001**

	Last feed before discharge taken exclusively from the breast,^f^ Yes (%)/No (%)	Last feed before discharge taken exclusively from the breast^f^					
		cOR	CI 95%	*p*	aOR	CI 95%	*p*
**Antenatal care provider** ^b^							
Obstetrician	235 897 (73.4%)/85 426 (26.6%)	Ref.			Ref.		
Midwife	176 157 (80.3%)/43 302 (19.7%)	1.47	1.45–1.49	**< 0.001**	1.23	1.21–1.25	**< 0.001**
General Practitioner (GP)	73 777 (82.4%)/15 704 (17.6%)	1.70	1.67–1.73	**< 0.001**	1.39	1.36–1.42	**< 0.001**
No/other intrapartum care provider	1597 (73.2%)/586 (26.8%)	0.99	0.90–1.09	0.786	1.08	0.96–1.22	0.189
**Lead intrapartum care provider**							
Obstetrician	288 849 (73.9%)/101 999 (26.1%)	Ref.			Ref.		
Midwife	166 450 (81.7%)/37 367 (18.3%)	1.57	1.55–1.59	**< 0.001**	1.23	1.21–1.25	**< 0.001**
General Practitioner (GP)	31 180 (84.6%)/5667 (15.4%)	1.94	1.89–2.00	**< 0.001**	1.68	1.62–1.73	**< 0.001**
No/other intrapartum care provider	2557 (84.6%)/465 (15.4%)	1.94	1.76–2.14	**< 0.001**	1.40	1.25–1.56	**< 0.001**
**Hospital type**							
Public	381 652 (78.2%)/106 423 (21.8%)	Ref.			Ref.		
Private	104 216 (73.0%)/38 641 (27.0%)	0.75	0.74–0.76	**< 0.001**	0.86	0.85–0.88	**< 0.001**
Private midwife	1418 (96.9%)/45 (3.1%)	8.79	6.53–11.82	**< 0.001**	3.71	2.61–5.28	**< 0.001**

*Note:* Bold values indicate statistical significance at the *p* < 0.05 level.

^a^ Adjusted for: maternal age, marital status, indigenous status, BMI, parity, smoking before 20 weeks of pregnancy, IRSD, area of living, country of birth, length of hospital stay (days), method of birth.

^b^ Attempted to breastfeed the baby or express breast milk at least once.

^c^ The discipline of the clinician who provided most occasions of antenatal care (VPDC manual).

^d^ The discipline of the clinician who, at the time of admission for the birth, is expected to be primarily responsible for making decisions regarding intrapartum care (VPDC manual).

^e^ Any infant formula given in hospital whether by bottle, cup, gavage or other means.

^f^ Last feed before discharge taken entirely from the breast, with no complementary feeding of any kind.

#### 3.2.2 Association Between Discipline of Intrapartum Care Provider and Early Breastfeeding

Women who had a midwife (aOR 1.15, 95% 1.12–1.19) or a GP (aOR 1.28, 95% CI 1.21–1.35) were more likely to initiate breastfeeding, compared to those who had an obstetrician as lead intrapartum provider. The adjusted odds of receiving formula in hospital were also lower for their infants (midwife: aOR 0.86, 95% CI 0.84–0.87; GP, aOR 0.70, 95% CI 0.68–0.72). Women who had a midwife (aOR 1.23, 95% CI 1.21–1.25) or GP (aOR 1.68, 95% CI 1.62–1.73) as lead intrapartum care provider had higher adjusted odds of giving the last feed before discharge entirely from the breast.

#### 3.2.3 Association Between Hospital Type and Early Breastfeeding

Women who gave birth in a private hospital were less likely to initiate breastfeeding (aOR 0.65, 95% CI 0.62–0.68) compared with women who gave birth in a public hospital, while women who gave birth assisted by a private midwife were markedly more likely to initiate breastfeeding (aOR 5.75, 95% CI 2.98–11.10). The odds of the infant receiving formula in hospital were higher for women who gave birth in a private hospital (aOR 1.34, 95% CI 1.32–1.37) compared with women who gave birth in a public hospital. Women who gave birth assisted by a private midwife had considerably lower adjusted odds (aOR 0.02, 95% CI 0.01–0.07) of the infant receiving formula in hospital. Women who gave birth in a private hospital had slightly lower adjusted odds (aOR 0.86, 95% CI 0.85–0.88) of giving the last feed before discharge entirely from the breast, while those who gave birth assisted by a private midwife had higher adjusted odds (aOR 3.71, 95% CI 2.61–5.28).

## 4 Discussion

Our findings demonstrate that mother/infant dyads are more likely to achieve better breastfeeding outcomes when they received antenatal care from a midwife or GP, compared to those who received care from an obstetrician. Similar patterns of findings regarding intrapartum care provider type were seen, where women with a GP or midwife as care provider obtained more favourable breastfeeding outcomes compared to those who received care from an obstetrician.

Our findings are consistent with results from The Infant Feeding Practices Survey II in the USA, which reported higher breastfeeding initiation in women receiving care from a midwife than those cared for by an obstetrician [18]. These findings may partly be explained by differences in women's intention to breastfeed, since previous research has shown that women seeking ANC from a midwife may demonstrate stronger intention to breastfeed than those receiving care from an obstetrician [19].

It is also important to consider that obstetricians assume responsibility for managing more complex pregnancies, particularly in public hospitals, which is known to adversely influence breastfeeding outcomes [20, 21, 22]. We were unable to adjust for any medical perinatal diagnoses (e.g., preeclampsia or gestational diabetes). Although we excluded mothers and infants admitted to intensive care and infants born preterm to reduce this potential bias, findings need to be interpreted with caution.

Another key finding was that women who gave birth in private hospitals were less likely to meet the WHO's breastfeeding recommendations, compared to those who gave birth in a public hospital. In contrast, the small number of women who gave birth assisted by a private midwife were significantly more likely to meet these recommendations, indicating that this model of care is beneficial in optimising breastfeeding outcomes. However, again, it is possible that medical and other characteristics that we did not have information on, and therefore were unable to adjust or analyse for, differed between these cohorts.

In our study, the most distinguished difference between private and public hospitals was observed to be the likelihood to initiate breastfeeding.

Before adjustment, we found a significant difference between private and public hospitals in terms of receiving formula in the hospital. However, when the adjustment factors *length of hospital stay* and *method of birth* were added to our model, this difference was greatly reduced. This suggests that the probability of receiving formula in hospital is also influenced by factors other than hospital type.

Meedya and colleagues used publicly available Australian data from New South Wales and Victoria from 2018 to 2019 to compare infant formula use between private and public hospitals [23]. They found a higher use of formula in non‐Baby Friendly Health Initiative (BFHI) private hospitals compared with non‐BFHI public hospitals in both states [23], which aligns with our results. The observed differences between the hospital types may be partly explained by their degree of adherence to the Baby Friendly Health Initiative (BFHI)'s programme [5]. At present, all accredited hospitals in Victoria are public hospitals [3], and it is thus likely that these birthing environments are more conducive to support breastfeeding.

Previous research has shown that individuals from higher socioeconomic groups have higher rates of breastfeeding than lower socioeconomic groups [24]. In our unadjusted analysis, we observed increased odds of breastfeeding initiation in private hospitals, which also serve a greater proportion of women from higher socioeconomic groups. However, after adjusting for influential factors, including socioeconomic status measured by IRSD, the odds were lower in private hospitals compared to public hospitals. Women from lower socioeconomic backgrounds are more likely to seek care at public hospitals [25] and there are likely other differences, including health related factors, between women seeking care at private versus public hospitals. Consequently, there is a range of possible confounding factors that may explain differences in breastfeeding outcomes between public and private care. Although our analyses adjusted for some of these, there may be other factors at play that we were unable to adjust for, which may have contributed to any residual confounding effects. Furthermore, the study period included 2020, during which the state of Victoria experienced some of the strictest COVID‐19 public health restrictions globally. These restrictions may have influenced maternity care, access to breastfeeding support, and infant feeding practices. We did not specifically evaluate the impact of the COVID‐19 pandemic on breastfeeding outcomes, and this warrants further investigation in future analyses. Finally, although two validation studies of the VPDC dataset demonstrated high agreement with medical records, discrepancies between actual infant feeding practices and their documentation may still exist. A strength of this study is that we included all women who gave birth to singleton term infants in Victoria, Australia, in 2010–2020, which substantially reduces the risk of selection bias. However, findings may have limited generalisability to other countries with different healthcare systems.

### 4.1 Research Implications

Breastfeeding is an important preventive public health intervention that can improve health outcomes for mothers and their children. However, unequal access to breastfeeding counselling and support can negatively affect these outcomes. It is vital that all maternity services in Victoria work towards Baby Friendly Hospital accreditation as there is strong evidence that infants born in Baby Friendly Hospitals have a longer duration of exclusive breastfeeding than other infants [26]. There is also a need to enhance breastfeeding education for obstetricians and other medical professionals [27, 28]. Future research could further explore differences in attitudes towards breastfeeding among various maternity care providers, their training in supporting women to breastfeed, and women's experiences of the advice they receive.

## Conflicts of Interest

The authors declare no conflicts of interest.

## Data Availability

The data that support the findings of this study are available on request from the corresponding author. The data are not publicly available due to privacy or ethical restrictions.

## Appendix 1

The following factors were adjusted for:

**Table:**

Factors	
Age group	< 20, 20–24, 25–29, 30–34, 35–39, 40 years or older
Marital status	In a couple relationship (married or de facto) or not in a couple relationship (never married, widowed, divorced, separated)
World region of birth of mother	Australia, Oceania and Antarctica, North‐West Europe, Southern and Eastern Europe, Americas, Sub‐Saharan Africa, North Africa and the Middle East, South‐East Asia, North‐East Asia, Southern and Central Asia
Indigenous status	Non‐Indigenous or Indigenous (Aboriginal or Torres Strait Islander origin)
Parity	Number of previous pregnancies that have resulted in a live or a stillbirth (of at least 20 weeks gestation or at least 400 g birth weight): 0, 1, 2, 3 or more
Area of living	Major cities of Australia, inner regional areas and outer regional‐, remote‐, and very remote (the last three combined into one group due to small group sizes)
Areas ranked due to relatively socio economic disadvantages	SEIFA: Index of Relative Socio‐Economic Disadvantage [IRSD] quantiles 1–5 [16]
Smoking status	Smoking habits during pregnancy before gestational age of 20 weeks, categorised as no smoking at all during pregnancy, quit smoking or continued smoking
Method of birth	Unassisted vaginal birth, vacuum extraction, forceps, planned caesarean—no labour, planned caesarean‐labour, unplanned caesarean—no labour, and unplanned caesarean‐labour
Length of hospital stay	Days, continuous variable
Body Mass Index (BMI)	Categorised based on the WHO classification [29] into underweight (< 18.5), normal weight (18.5–24.9), pre‐obesity (25.0–29.9), obesity Class I (30.0–34.9), obesity Class II (35–39.9), and obesity Class III (≥ 40).
